# Neonatal Enterovirus Myocarditis With Severe Dystrophic Calcification: Novel Treatment With Pocapavir

**DOI:** 10.1177/2324709617729393

**Published:** 2017-09-14

**Authors:** Samuel G. Wittekind, Catherine C. Allen, John L. Jefferies, Mantosh S. Rattan, Peace C. Madueme, BreAnn N. Taylor, Ryan A. Moore

**Affiliations:** 1 Cincinnati Children’s Hospital Medical Center and University of Cincinnati, Cincinnati, OH, USA

**Keywords:** myocarditis, cardiomyopathy, heart failure, computed tomography, echocardiography

## Abstract

Dystrophic myocardial calcification occurs in the setting of myocardial injury and normal serum calcium. We present a case of a neonate with prominent dystrophic calcification and severe left ventricular systolic dysfunction in the setting of enterovirus myocarditis. These findings are superbly illustrated by multiple imaging modalities. The patient was treated with the novel antiviral, pocapavir, in addition to a standard heart failure regimen. The dystrophic calcification persisted but the left ventricle remodeled significantly. To our knowledge, this is the first reported use of pocapavir for this indication. The literature regarding enterovirus myocarditis and pocapavir is briefly reviewed.

## Introduction

Neonatal enterovirus myocarditis is a rare and often fatal disease. In reported cases, those who survived the acute inflammatory phase developed chronic heart failure, aneurysm formation within the left ventricle (LV), and mitral regurgitation requiring chronic medical therapy.^1^ Dystrophic calcifications occur in the setting of a normal serum calcium and myocardial injury while metastatic calcifications occur in viable tissue due to impaired calcium-phosphorus metabolism. The mechanism of dystrophic calcification is not completely understood.^2^ The presence of dystrophic myocardial calcification in the neonatal period suggests an in utero insult. In most cases the calcifications persist despite clinical improvement. Treatment options are limited; heart transplantation for necrotic enterovirus myocarditis has been reported.^3^ Successful treatment of neonatal enterovirus myocarditis with antiviral medication has not been reported in the literature. Pocapavir, an investigational oral antiviral, has been used in a case of neonatal enterovirus sepsis, but myocarditis was not a prominent feature in this report.^4^

## Case Report

**Figure 1 and Movie 1.**
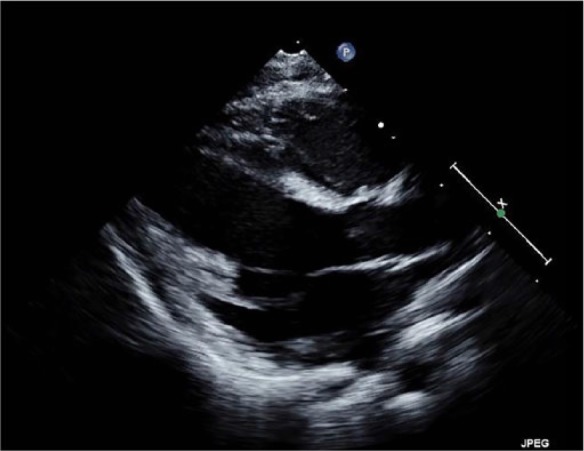
Transthoracic echocardiogram (parasternal long axis view at end-diastole), prior to pocapavir treatment, demonstrated a hyperechoic basal and mid-ventricular septum, papillary muscle, and posterior left ventricular (LV) wall; LV systolic function was severely depressed.

**Figure 2.**
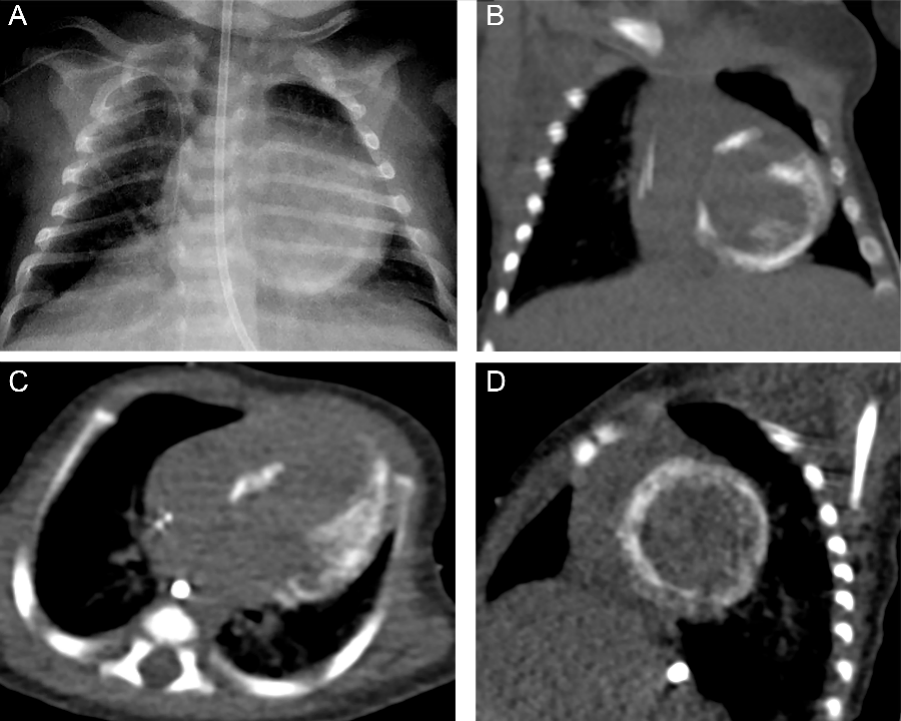
(A) Chest radiograph anterior-posterior view demonstrated dystrophic calcification of left ventricular (LV) myocardium and papillary muscle 3 weeks into illness. The affected LV myocardium corresponded to hyperechoic ventricular segments seen on echocardiography. (B) Noncontrast computed tomography (CT) coronal view. (C) Noncontrast CT axial view. (D) Noncontrast CT reformatted short axis view.

A 2-week-old female born at 34 weeks following a pregnancy complicated by maternal drug use and hepatitis C presented with fever, lethargy, coffee ground emesis, petechiae, and jaundice. She was found to have hepatitis and coagulopathy with thrombocytopenia requiring multiple transfusions. Empiric antibiotics and acyclovir were started. The initial transthoracic echocardiogram demonstrated a left ventricular end-diastolic diameter (LVEDD) of 2.2 cm (*z* score = +1.19), left ventricular ejection fraction (LVEF; bullet method) of 30% to 35%, and hypokinesis of the mid posterolateral and basal anteroseptal LV segments with increased echogenicity of these segments and the anterolateral papillary muscle. A repeat echocardiogram 4 days later demonstrated interval worsening with a LVEF of 25% to 30% with akinesis and hyperechogenicity of the same LV segments (Figure 1, Movie 1). The movies are available online at http://journals.sagepub.com/home/hic. Chest radiograph suggested myocardial calcification (Figure 2A) and noncontrast computed tomography (CT) of the chest confirmed the diagnosis of dystrophic calcification of LV myocardium (Figure 2B-D). Serum calcium levels were normal.

Polymerase chain reaction (PCR) for enterovirus was positive from blood, stool, urine, and nasal samples. Hepatitis C, cytomegalovirus, and human immunodeficiency serum PCR tests were negative. Myocarditis was treated with 2 g/kg of intravenous immunoglobulin and steroids. Endomyocardial biopsy was not performed because clinical suspicion was sufficiently high that enterovirus was the causative agent of myocarditis. One week after presentation, oral pocapavir^4^ was administered on a compassionate basis at a dose of 162 mg/day. Eleven of 14 days of treatment were completed. This was because the patient was made temporarily nil by mouth after a single bloody stool on day 2 of treatment. There were no further episodes of hematochezia. Stool PCR for enterovirus remained positive until 1 month posttreatment; viremia persisted for 2 months. The consulting infectious disease service did not recommend immunodeficiency testing. The liver function normalized after antiviral treatment. Heart failure symptoms were initially treated with diuretics and a milrinone infusion. Steroids were administered for frequent ventricular ectopy, which resolved prior to discharge. She was transitioned to an oral heart failure regimen of carvedilol, captopril, spironolactone, furosemide, and aspirin then discharged home approximately 3 months later. At the time of discharge the serum brain natriuretic peptide (BNP) was between 400 and 500 pg/mL and the LVEDD was 3.6 cm with an LVEF of 30% to 35%.

**Figure 3 and Movie 2.**
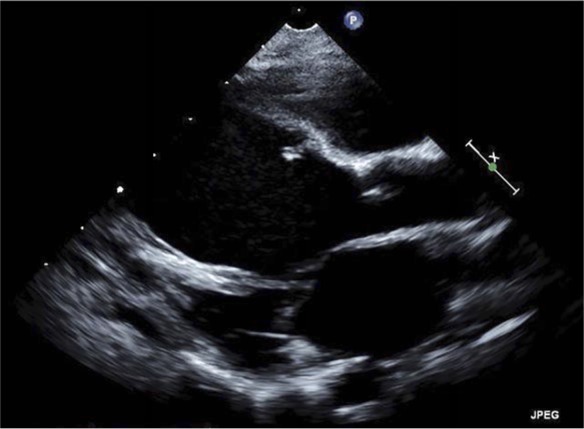
Two-year follow-up transthoracic echocardiogram (parasternal long axis view at end-diastole) demonstrated myocardial thinning with left ventricular (LV) dilation but overall decrease in echogenicity; LV systolic function was moderately depressed.

Serial echocardiograms have demonstrated a dilated cardiomyopathy. Two years after presentation, the LVEDD increased to 4.4 cm (*z* score = +6.5) and the left atrium was severely dilated (indexed left atrial volume = 50.2 mL/m^2^). The LVEF was 40% with moderate mitral regurgitation. The basal septum, entire posterolateral wall, and papillary muscles remained hyperechoic and hypokinetic, and the right ventricular systolic function remained normal (Figure 3, Movie 2). The serum BNP normalized to 141 pg/mL. Nearly 3 years after presentation, the calcifications persisted but LV had further remodeled; the LVEDD decreased to 4.21 cm (*z* score = +5) and the LVEF increased to 44%. Since her initial illness she has been hospitalized just once for 3 days due to respiratory syncytial virus infection. At the present time she remains asymptomatic off diuretic therapy.

## Discussion

Pocapavir, also known as SCH 48973 and V-073, is a potent, selective anti-enteroviral agent only available as an emergency investigational drug. The drug belongs to a picornavirus antiviral mechanistic class called capsid inhibitors that block virus uncoating and viral RNA release into cells, which in turn prevents virus replication. Pocapavir is administered orally and is highly protein-bound and excreted exclusively in feces.^5^ Pocapavir was originally created to treat poliovirus, but was found to also have variable activity against 15 of the most commonly isolated enterovirus subtypes.^6^ Surveillance data from the Center for Disease Control indicate these 15 subtypes compose 65% to 89% of the recovered enterovirus isolates in the United States and include echovirus types 3 to 7, 9, 11, 24, and 30; Coxsackie group A9 and groups B1, B2, B3, B4, and B5.^7^ Our institute’s internal PCR test used in this case was designed to detect any enterovirus and has been validated by positive detection of the following: Coxsackie groups A9, A24, B5, B6; and echovirus groups 4, 9, 11, 25, 68, 71.

Noninvasive imaging was crucial in the diagnosis and management of this case of enterovirus myocarditis with dystrophic calcification. Echocardiography raised suspicion for myocardial calcification, which was confirmed by CT. These imaging modalities illustrated the hallmark features of the disease process: hyperechoic and radiopaque myocardial segments with impaired cardiac function. While this patient did not escape the sequela of dilated cardiomyopathy and need for chronic heart failure therapy, the case is notable for the observed resolution of hepatitis, eventual viral clearance, LV reverse-remodeling, and excellent outcome seen with pocapavir treatment. Our experience is promising and further investigation is warranted. In future cases of enterovirus myocarditis, identifying the particular viral species involved might help determine which enteroviruses are associated with myocardial dystrophic calcification and which may respond to antiviral treatment with pocapavir.

## References

[1] Freund MW Kleinveld G Krediet TG Van Loon AM Verboon-Maciolek MA Prognosis for neonates with enterovirus myocarditis. Arch Dis Child Fetal Neonatal Ed. 2010;95:F206-F212. 20444813 10.1136/adc.2009.165183

[2] Mana M Sanguineti F Unterseeh T Bouvier E Garot J Petrified myocardium. The age of stone? Circulation. 2012;126:1139-1142. 22927476 10.1161/CIRCULATIONAHA.112.100321

[3] Simmonds J Cubitt D Ashworth M Burch M Successful heart transplantation following neonatal necrotic enterovirus myocarditis. Pediatr Cardiol. 2008;29:834-837. 18158605 10.1007/s00246-007-9182-z

[4] Torres-Torres S Myers AL Klatte JM First use of investigational antiviral drug pocapavir (V-073) for treating neonatal enteroviral sepsis. Pediatr Infect Dis J. 2015;34:52-54. 25229269 10.1097/INF.0000000000000497

[5] Oberste MS Moore D Anderson B Pallansch MA Pevear DC Collett MS In vitro antiviral activity of V-073 against polioviruses. Antimicrob Agents Chemother. 2009;53:4501-4503. 19635956 10.1128/AAC.00671-09 PMC2764203

[6] Buontempo PJ Cox S Wright-Minogue J SCH 48973: a potent, broad-spectrum, antienterovirus compound. Antimicrob Agents Chemother. 1997;41:1220-1225. 9174174 10.1128/aac.41.6.1220 PMC163890

[7] Strikas RA Anderson LJ Parker RA Temporal and geographic patterns of isolates of nonpolio enterovirus in the United States, 1970-1983. J Infect Dis. 1986;153:346-351. 3003207 10.1093/infdis/153.2.346

